# Porphyrin-lipid stabilized paclitaxel nanoemulsion for combined photodynamic therapy and chemotherapy

**DOI:** 10.1186/s12951-021-00898-1

**Published:** 2021-05-25

**Authors:** Enling Chang, Jiachuan Bu, Lili Ding, Jenny W. H. Lou, Michael S. Valic, Miffy. H. Y. Cheng, Véronique Rosilio, Juan Chen, Gang Zheng

**Affiliations:** 1 Princess Margaret Cancer Centre, University Health Network, PMCRT 5-353, 101 College Street, Toronto, ON M5G 1L7 Canada; 2 Institute of Biomedical Engineering, University of Toronto, PMCRT 5-354, 101 College Street, Toronto, ON M5G 1L7 Canada; 3 Department of Medical Biophysics, University of Toronto, Toronto, Canada; 4 Institut Galien Paris-Saclay, Université Paris-Saclay, CNRS, Châtenay-Malabry, France

**Keywords:** Photodynamic therapy, Porphyrin, Chemotherapy, Paclitaxel, Nanoparticle, Drug delivery, Cancer therapy

## Abstract

**Background:**

Porphyrin-lipids are versatile building blocks that enable cancer theranostics and have been applied to create several multimodal nanoparticle platforms, including liposome-like porphysome (aqueous-core), porphyrin nanodroplet (liquefied gas-core), and ultrasmall porphyrin lipoproteins. Here, we used porphyrin-lipid to stabilize the water/oil interface to create porphyrin-lipid nanoemulsions with paclitaxel loaded in the oil core (PLNE-PTX), facilitating combination photodynamic therapy (PDT) and chemotherapy in one platform.

**Results:**

PTX (3.1 wt%) and porphyrin (18.3 wt%) were loaded efficiently into PLNE-PTX, forming spherical core–shell nanoemulsions with a diameter of 120 nm. PLNE-PTX demonstrated stability in systemic delivery, resulting in high tumor accumulation (~ 5.4 ID %/g) in KB-tumor bearing mice. PLNE-PTX combination therapy inhibited tumor growth (78%) in an additive manner, compared with monotherapy PDT (44%) or chemotherapy (46%) 16 days post-treatment. Furthermore, a fourfold reduced PTX dose (1.8 mg PTX/kg) in PLNE-PTX combination therapy platform demonstrated superior therapeutic efficacy to Taxol at a dose of 7.2 mg PTX/kg, which can reduce side effects. Moreover, the intrinsic fluorescence of PLNE-PTX enabled real-time tracking of nanoparticles to the tumor, which can help inform treatment planning.

**Conclusion:**

PLNE-PTX combining PDT and chemotherapy in a single platform enables superior anti-tumor effects and holds potential to reduce side effects associated with monotherapy chemotherapy. The inherent imaging modality of PLNE-PTX enables real-time tracking and permits spatial and temporal regulation to improve cancer treatment.

**Graphic Abstract:**

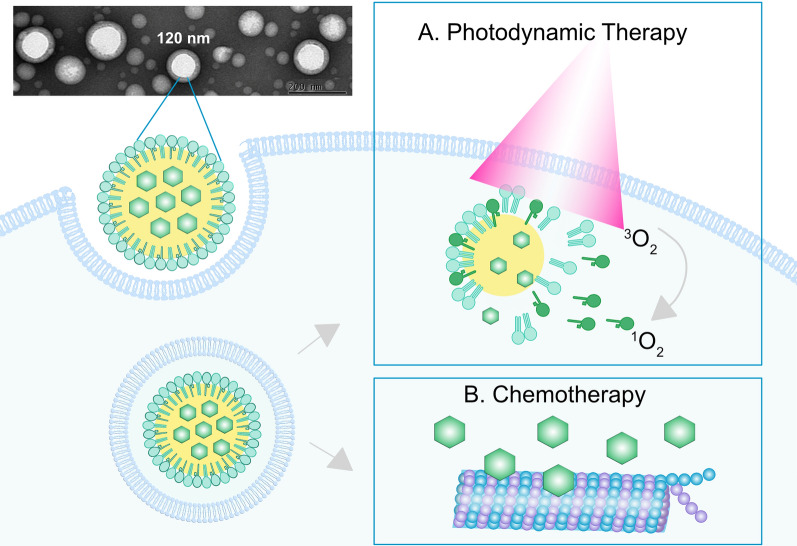

**Supplementary Information:**

The online version contains supplementary material available at 10.1186/s12951-021-00898-1.

## Introduction

Photodynamic therapy (PDT) is a minimally-invasive treatment modality, in which irradiation of photosensitizers that have accumulated in the tumor can produce cytotoxic reactive oxygen species to induce tumor regression. Subsequently, a cascade of therapeutic effects occurs, including apoptosis and necrosis of tumor cells, tumor vasculature damage or permeabilization, reduction in supply of oxygen and essential nutrients, and activation of the immune system responses to induce tumor regression [1–3]. However, PDT as a monotherapy often suffers from incomplete tumor killing due to limited light penetration in tissue (1–3 mm depth for red light) and challenges in treating area where light cannot reach or cancers that have spread to many places [4].

Systemic administration of chemotherapy can effectively eradicate tumors, but often causes serious side effects that reduce the quality of life of patients. Several studies demonstrated that PDT can enhance drug delivery by increasing the permeability of tumor vasculature [5–7], decreasing solid tissue stress [8], reducing interstitial fluid pressure [9] and decreasing tumor stemness [10]. Therefore, the combination of PDT and chemotherapy in a single platform may provide a treatment strategy that enables synergistic therapeutic effects, reduces side effects, and minimizes multi-drug resistance [11–15]. Light controlled PDT enables localized treatment of malignant tissues with minimal damage of underlining connective tissues, whereas chemotherapy can eliminate surviving tumor cells, especially for deep-seated tumors that lasers have difficulty penetrating. Furthermore, PDT enables vascular permeabilization and reduces extravascular barriers, which may enhance the deposition of chemotherapeutic drugs into tumors to improve therapeutic efficacy, while limiting systemic off-target toxicities.

Paclitaxel (PTX), approved by the FDA for the first-line treatment of breast, pancreatic, ovarian, Kaposi's sarcoma, and non-small cell lung cancers, is a microtubule-stabilizing drug that causes abnormal mitosis in tumors [16]. However, its poor aqueous solubility (< 0.01 mg/ml) hinders its effective circulation and subsequent tumor accumulation [17]. In addition, PTX is highly toxic and induces several clinical side effects, including hair loss, bone marrow suppression, allergic reactions, and diarrhea [18]. In 2000, Taxol was developed to enhance PTX’s solubility in physiological solution, but cremophor EL, a stabilizing agent in the formulation, triggered hypersensitivity reactions [19]. To overcome these issues, Abraxane, a nanosized cremophor EL-free albumin-bound PTX was developed in 2005 and demonstrated enhanced delivery, reduced hypersensitivity reactions, and better overall treatment response and survival compared to Taxol [20]. However, toxicities concern still remain in incidence of neutropenia and neurotoxicity [21–24]. Thus, it is essential to develop an improved PTX delivery nanoplatform for safe and efficient delivery.

Porphyrins and their derivatives have been clinically approved as efficacious PDT agents for treatment in lung, esophageal, bile duct, bladder, ovarian, and cervical cancers [25, 26]. We recently developed highly stable, porphyrin salt-stabilized nanoemulsions capable of loading PTX in a oil core [27]. By integrating porphyrin (on the shell) and PTX (in the core) in a single nanoparticle, the porphyrin nanoemulsion demonstrated additive antitumor efficacy. However, this porphyrin salt nanoemulsion exhibited unsatisfactory tumor accumulation (< 1 ID %/g), thus requiring a high drug dose (PTX 7.2 mg/kg, Pyro-salt 30 $\mu$ mol/kg) for effective treatment.

**Fig. 1:**
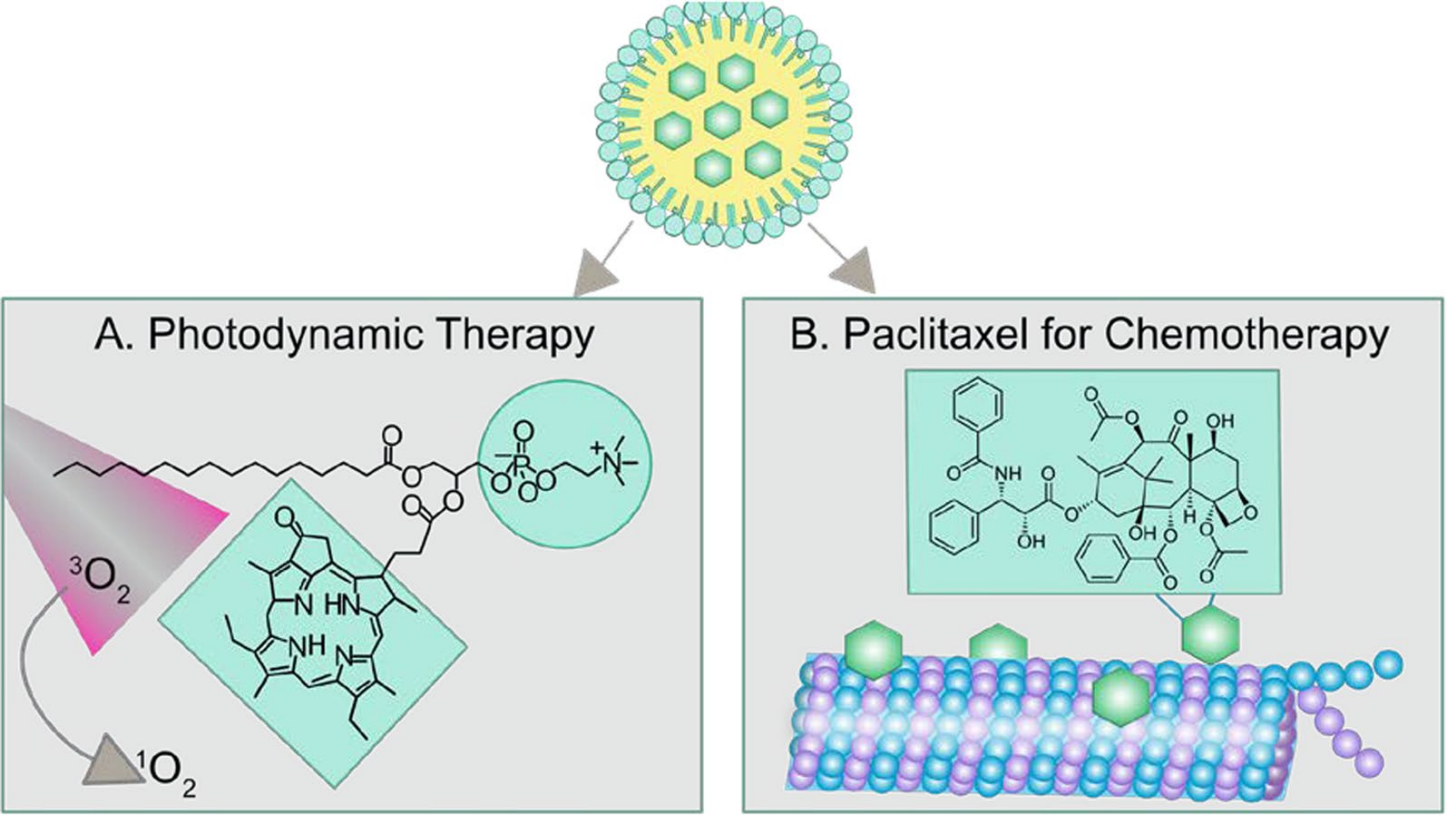
Porphyrin-lipid stabilized nanoemulsions with paclitaxel loaded in the oil core (PLNE-PTX) integrates photodynamic therapy and chemotherapy in one platform

Porphyrin-lipids as building blocks perform well in nanoparticles construction for cancer multimodal imaging and therapeutics [28]. Beside the PDT reactivity, the intrinsic fluorescence of porphyrin can be used for tracking of nanoparticle delivery. Various metals such as radioisotopes (e.g. ^64^Cu) and paramagentic metal  (e.g. Mn) can be robustly chelated into porphyrin ring to allow positron emission tomography (PET) and magnetic resonance imaging (MRI), respectively [29–31]. As a fully organic molecule porphyrin-lipid is enzymatically biodegradable in vivo. Administration of porphyrin-lipid at doses of 1000 mg/kg elicited minimal acute toxicities in mice [28, 32]. We have previously used porphyrin-lipid as a building block to create several multimodal nanoparticle platforms, including liposome-like porphysomes (aqueous-core) [28], porphyrin nanodroplets (liquefied gas-core) [33], and ultrasmall porphyrin lipoproteins [34, 35]. Herein, we utilized porphyrin-lipid to stabilize water/oil interface to create porphyrin-lipid nanoemulsion with paclitaxel loaded in the oil core (PLNE-PTX). The resultant PLNE-PTX integrated high amounts of a chemotherapeutic agent and a photosensitizer in a single nanoparticle and performed excellently in drugs delivery in vivo (Fig. 1). PLNE-PTX-enabled combination of PDT and chemotherapy demonstrated superior therapeutic efficacy to single PDT and single chemotherapy in KB tumor-bearing mice.

## Results

### Synthesis and characterization of a porphyrin-lipid nanoemulsion

**Fig. 2:**
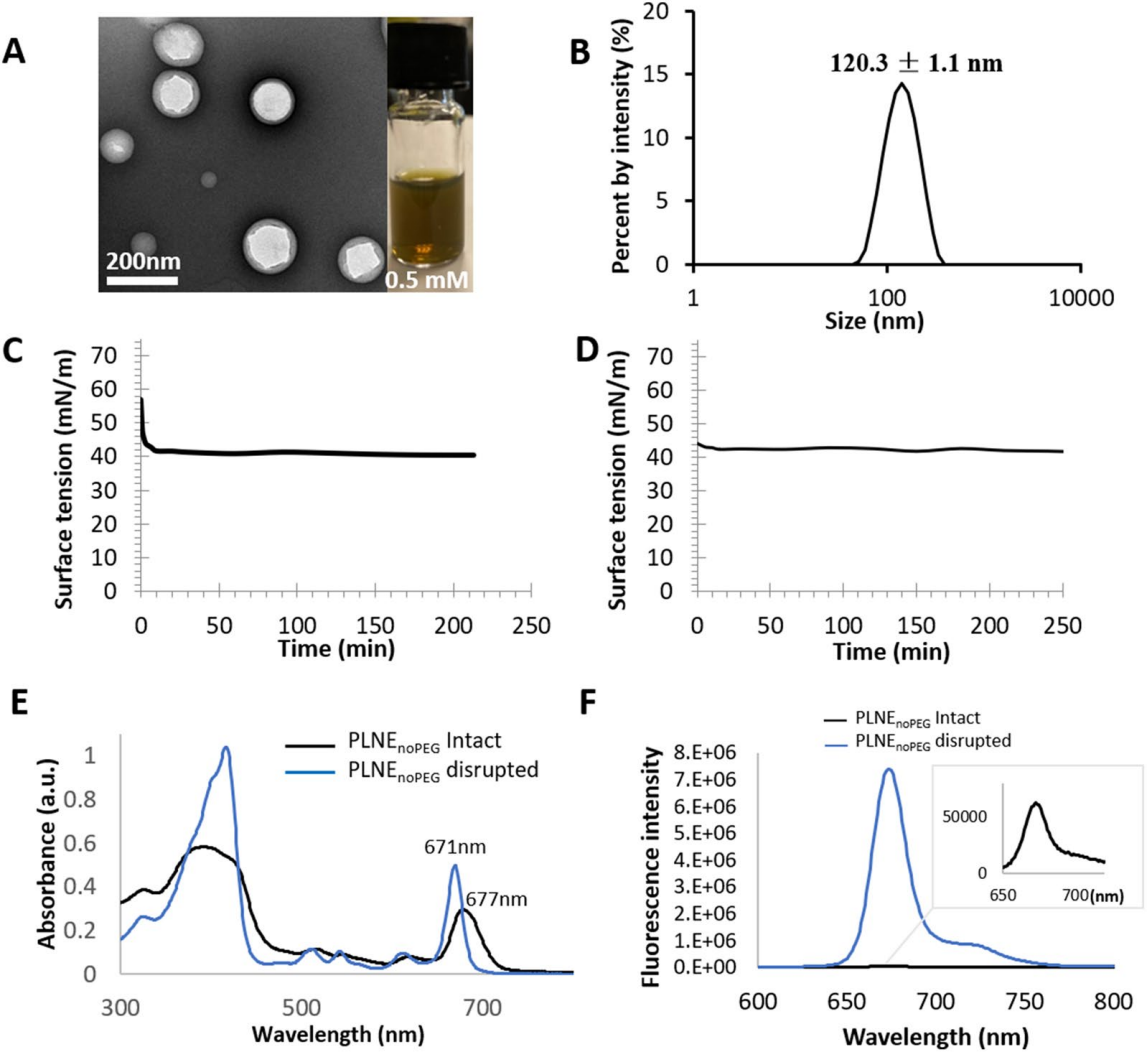
**A** TEM and photography images of PLNE_noPEG_. **B** DLS size distribution of PLNE_noPEG_. **C** Surface tension was measured on day 15 (D15); **D** Surface tension was measured after 40 days following the first measurements (D55). **E** UV–vis spectrum and **F** fluorescence spectrum of intact PLNE_noPEG_ (ddH_2_O) or disrupted PLNE_noPEG_ (1% Triton in ddH_2_O)

Porphyrin-lipid is amphiphilic, as the phosphate head group facilitates strong hydrophilicity, while the hydrocarbon-chain tail confers hydrophobicity. This amphiphilicity was leveraged, to create a nanoemulsion shell that stabilized an oil core for hydrophobic drug loading. A porphyrin-lipid nanoemulsion was formulated by the self-assembly of porphyrin-lipid around a glyceryl trioctanoate oil core, resulting in a simple, monodisperse, two-component nanoemulsion (PLNE_noPEG_) with a hydrodynamic diameter of 120.3 ± 1.1 nm and surface charge of − 2.4 ± 0.3 mV (Fig. 2A and B). Compared to the previous nanoemulsion composed of porphyrin-salt which had a zeta potential of − 25 ± 1.1 mV [27], our new formulation had a porphyrin-lipid shell, which resulted in a neutral surface (zeta potential − 2.4 ± 0.3 mV) instead. The surface tension of PLNE_noPEG_ was measured after two weeks storage, on day 15 (D15), and 40 days following the first measurement (D55). The equilibrium of surface tension was obtained in less than 20 min to 40–42 mN/m at D15 (Fig. 2C), and immediately at 42.8 mN/m at D55 (Fig. 2D). These data suggested that the porphyrin-lipid rapidly stabilized the interface. Meanwhile, UV–Vis absorption spectrum of PLNE_noPEG_ showed a slight red shift in its absorption spectra (6 nm) relative to the porphyrin-lipid monomer (Fig. 2E). PLNE_noPEG_ had high fluorescence quenching (> 98%) (Fig. 2F), indicating suppressed photodynamic reactivity in intact PLNE according to previous finding of direct correlation between fluorescence and singlet oxygen generation in porphyrin-lipid based nanostructures [28, 34].

**Fig. 3:**
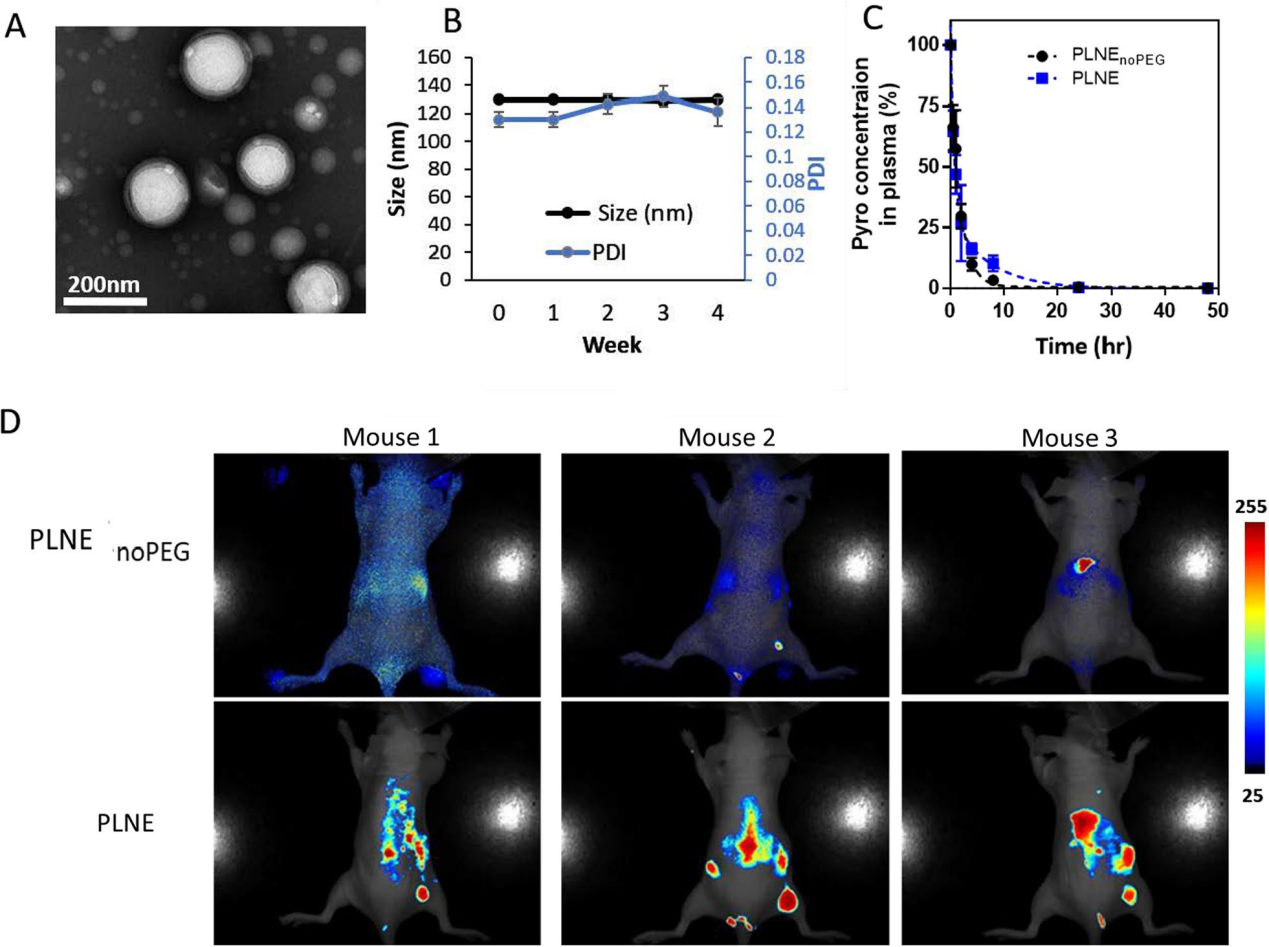
**A** TEM image of PLNE containing 5% of DSPE-PEG 2000 in the nanoemulsion construction. **B** Size and PDI stability of PLNE in ddH_2_O at 4 °C. **C** The blood circulation profile of PLNE_noPEG_ compared to PLNE. **D** In vivo fluorescence imaging at 24 h post injection of PLNE_noPEG_ relative to PLNE

To create a more stable formulation, 5% mol 1,2-distearoyl-sn-glycero-3-phosphoethanolamine-N-[amino (polyethylene glycol)-2000] (DSPE-PEG 2000) was incorporated into the nanoemulsion (PLNE). Relative to PLNE_noPEG_, PLNE had a similar hydrodynamic diameter (119.7 ± 0.6 nm) (Fig. 3A) and surface charge (zeta potential −1.84 ± 0.6 mV). PLNE showed good stability at 4 °C, as evidenced by minimal changes in size and polydispersity index (PDI) after 4 weeks of storage (Fig. 3B). Notably, PEGylation prolonged the plasma circulation of PLNE. PLNE_noPEG_ had a half-life of 2.50 ± 1.54 h, whereas PLNE had a longer half-life of 3.67 ± 0.22 h (Fig. 3C). Moreover, the intrinsic fluorescence of porphyrin-lipid enabled fluorescence tracking of the nanoemulsions’ biodistribution and activation after administration. Both in vivo nd ex vivo fluorescence imaging demonstrated enhanced tumor accumulation of PLNE relative to PLNE_noPEG_ (Fig. 3D, Additional file 1: Figure S1). As PEGylation improved stability of porphyrin-lipid nanoemulsions, this formulation was used in subsequent therapeutic studies.

### PLNE-PTX formulation optimization

**Fig. 4:**
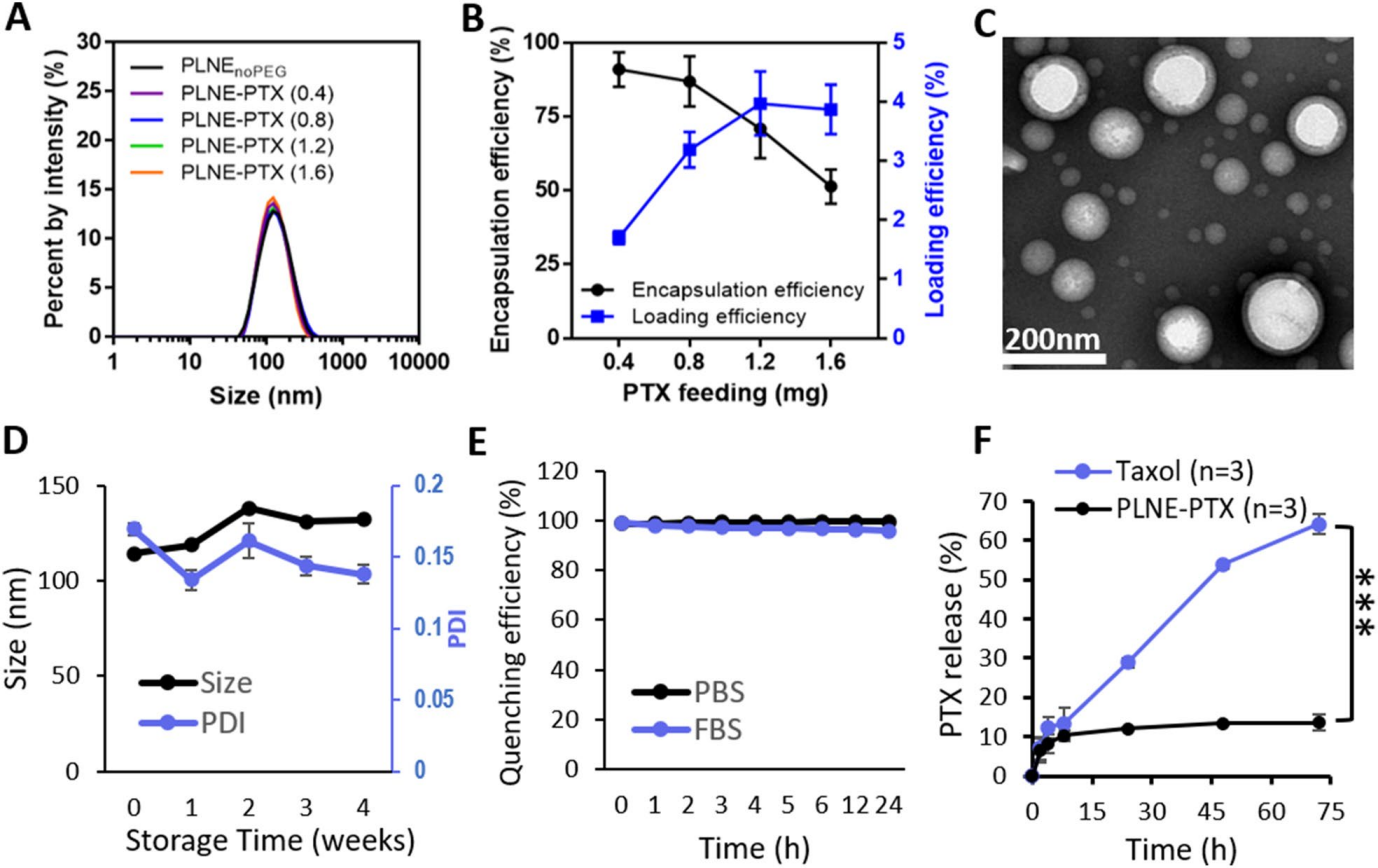
**A** DLS size distribution spectrum of PLNEs fed with varying amounts of PTX. **B** PTX encapsulation and loading efficiencies for various formulations of PLNE-PTX. **C** TEM image of the optimized PLNE-PTX formulation. **D** Stability of PLNE-PTX as assessed by changes in size and PDI in ddH_2_O at 4 °C. **E** Serum stability of PLNE-PTX at 37 °C for 24 h in 50% FBS or DPBS, assessed by fluorescence quenching efficiency. **F** PTX release from PLNE-PTX relative to the commercially available Taxol was evaluated by PTX leakage study at 37 °C (*** p < 0.001, n = 3 per group)

To investigate the drug loading capacity of PLNE, varying amounts of PTX was fed into PLNE and resulting formulations were characterized (PLNE-PTX). The formulation components detail for various PLNEs are listed in Table 1. As shown in Table 2 and Fig. 4A, feeding PTX (from 0.4 to 1.6 mg) into PLNE had negligible effects on particle size (Z-average: 115–122 nm) and monodispersity (PDI: 0.13–0.15). Of the various feeding amounts, the 0.8 mg PLNE-PTX had a high PTX encapsulation efficiency of 87.0 ± 8.4% and acceptable PTX loading (3.1 ± 0.3 wt% of particle) (Fig. 4B), along with a high density of porphyrin-lipid in the shell (18.3 ± 0.1 wt% of particle). Therefore, this optimized 0.8 mg PLNE-PTX formulation was selected for further investigation.

**Table 1:** Formulation of PLNE_noPEG_, PLNE, and 0.4–1.6 mg PTX-PLNE

	PLNE_noPEG_	PLNE	0.4 mg PLNE-PTX	0.8 mg PLNE-PTX	1.2 mg PLNE-PTX	1.6 mg PLNE-PTX
Porphyrin-lipid	4.1 mg (4 µmol)					
DSPE-PEG 2000	n/a	0.58 mg (0.2 µmol)				
Glyceryl trioctanoate	19.2 mg (40.8 µmol)					
Paclitaxel	n/a		0.4 mg (0.5 µmol)	0.8 mg (0.9 µmol)	1.2 mg (1.4 µmol)	1.6 mg (1.9 µmol)

**Table 2:** Size and PDI of PLNE_noPEG_, PLNE, and 0.4–1.6 mg PTX-PLNE

	PLNE_noPEG_	PLNE	0.4 mg PLNE-PTX	0.8 mg PLNE-PTX	1.2 mg PLNE-PTX	1.6 mg PLNE-PTX
Size (nm)	120.3 ± 1.1	119.7 ± 0.6	115.2 ± 0.4	118.9 ± 0.7	117.3 ± 0.5	112.2 ± 0.4
PDI	0.17 ± 0.016	0.13 ± 0.006	0.12 ± 0.009	0.13 ± 0.007	0.14 ± 0.004	0.14 ± 0.003

### Optical properties and stability of PLNE-PTX

Transmission electron microscopy (TEM) revealed that the optimized PLNE-PTX were monodisperse, with a spherical shell-core structure and ~ 120 nm in diameter (Fig. 4C). Similar to the empty PLNE, PLNE-PTX exhibited a slight Q band red-shifted in the UV–vis absorption spectra (Additional file 1: Figure S2A). Meanwhile, porphyrin-lipid fluorescence was highly quenched in intact particles (98.6 ± 3.2% quenching efficiency) but was restored efficiently upon disruption of the nanoemulsion (Additional file 1: Figure S2B). The size of PLNE-PTX remained similar at ~ 120 nm with minimal changes to PDI (~ 0.15) after 4 weeks storage at 4 °C (Fig. 4D). After incubation with 50% FBS at 37 °C for 24 h, fluorescence quenching of PLNE-PTX remained high (96–99%), indicating robust stability in serum-like conditions (Fig. 4E). The stability of PTX in PLNE-PTX was assessed viaPTX leakage by dialysis at 37 °C and compared with commercially available Taxol. As shown in Fig. 4F, Taxol rapidly diffused through a dialysis tube to the surrounding DPBS solution: 64.2 ± 2.7% of PTX leaked within 72 h. In contrast, PLNE-PTX exhibited a slower PTX leakage profile with only 13.5 ± 1.9% of PTX leaked in the same duration (4.8-fold reduction). This delayed release of PTX by PLNE-PTX may prevent unwanted PTX leakage into the circulatory system and enable greater amounts of PTX to accumulate in the tumor.

### Intracellular uptake and therapeutic evaluation of PLNE-PTX in vitro

**Fig. 5:**
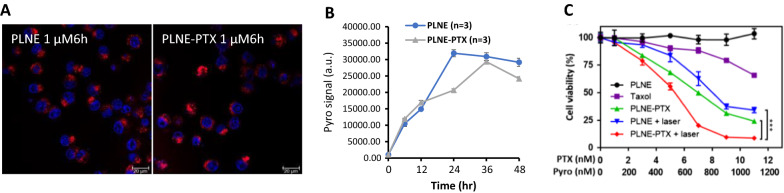
**A** Intracellular uptake of PLNE and PLNE-PTX was visualized by confocal microscopy (scare bar = 20 µm). **B** The cellular uptake of PLNE and PLNE-PTX was quantified (n = 3 per group) by flow cytometry. **C** In vitro therapeutic efficacy was deteremined using an alamarBlue assay. KB cells were treated with either: (1) PLNE, (2) monotherapy Taxol, (3) PLNE-PTX single chemotherapy, (4) PLNE + laser irradiation for monotherapy PDT, and (5) PLNE-PTX + laser irradiation for combination therapy. %) (*** represents p < 0.001 by two-way ANOVA test; n = 6 per group)

The intrinsic fluorescence of porphyrin-lipid enabled visualization of the intracellular uptake of PLNE by fluorescence microscopy. As shown in Fig. 5A, PLNE were internalized into tumor cells rapidly. Their intracellular uptake profile was further investigated by time-dependent uptake, as quantified by flow cytometry. As shown in Fig. 5B, porphyrin-lipid fluorescence associated with KB cells increased over time, and plateaued at 24 h, indicative of intracellular PLNE-PTX accumulation and activation. After 24 h incubation, PLNE-PTX were disrupted, as such, monomeric porphyrin-lipid facilitated fluorescence imaging and PDT. Therefore, a 24 h incubation period was selected for subsequent in vitro PDT treatment. As no significant difference in intracellular uptake was observed between PLNE and PLNE-PTX, it was concluded that incorporation of PTX into PLNE did not influence the platform’s intracellular uptake. Accordingly, porphyrin-lipid fluorescence can also serve as a proxy for intracellular delivery of PTX.

The therapeutic efficacy of PLNE-PTX in combination PDT and chemotherapy was evaluated with an alamarBlue assay. Taxol was used as a drug control for monotherapy PTX and empty PLNE was used for monotherapy PDT. As shown in Fig. 5C, KB tumor cells incubated with PLNE at porphyrin-lipid concentrations ranging from 0 to 1100 nM remained viable (98–100% cell viability), indicative of the safety of PLNE as a drug delivery vehicle. After laser irradiation (671 nm, 10 J/cm^2^), cell death occurred, with the magnitude corresponding to PLNE concentration. At a porphyrin-lipid concentration of 1100 nM, KB cell viability decreased to 34.22 ± 2.4%. Meanwhile, PLNE-PTX showed dark toxicity, which was attributed to the chemotherapeutic effect of PTX. At a PTX concentration of 11 nM, PLNE-PTX decreased cell viability to 24.22 ± 0.74% while Taxol at the same concentration reduced cell viability of 65.77% ± 1.86%. The enhanced chemotherapeutic efficacy by PLNE-PTX may arise from improved intracellular uptake of PTX by nanoemulsion delivery. Upon laser irradiation, PLNE-PTX (1100 nM porphyrin-lipid, 11 nM PTX) enabled combination PDT and chemotherapy, which further decreased KB cell viability to 8.75 ± 1.1%. As a result, PLNE-PTX elicited a significantly enhanced anti-tumor response, compared to monotherapy chemotherapy and monotherapy PDT by 7.5-fold and 3.9-fold, respectively (*** represents p < 0.001 by two-way ANOVA test; n = 6 per group).

### In vivo pharmacokinetics and tumor accumulation of PLNE-PTX

**Fig. 6:**
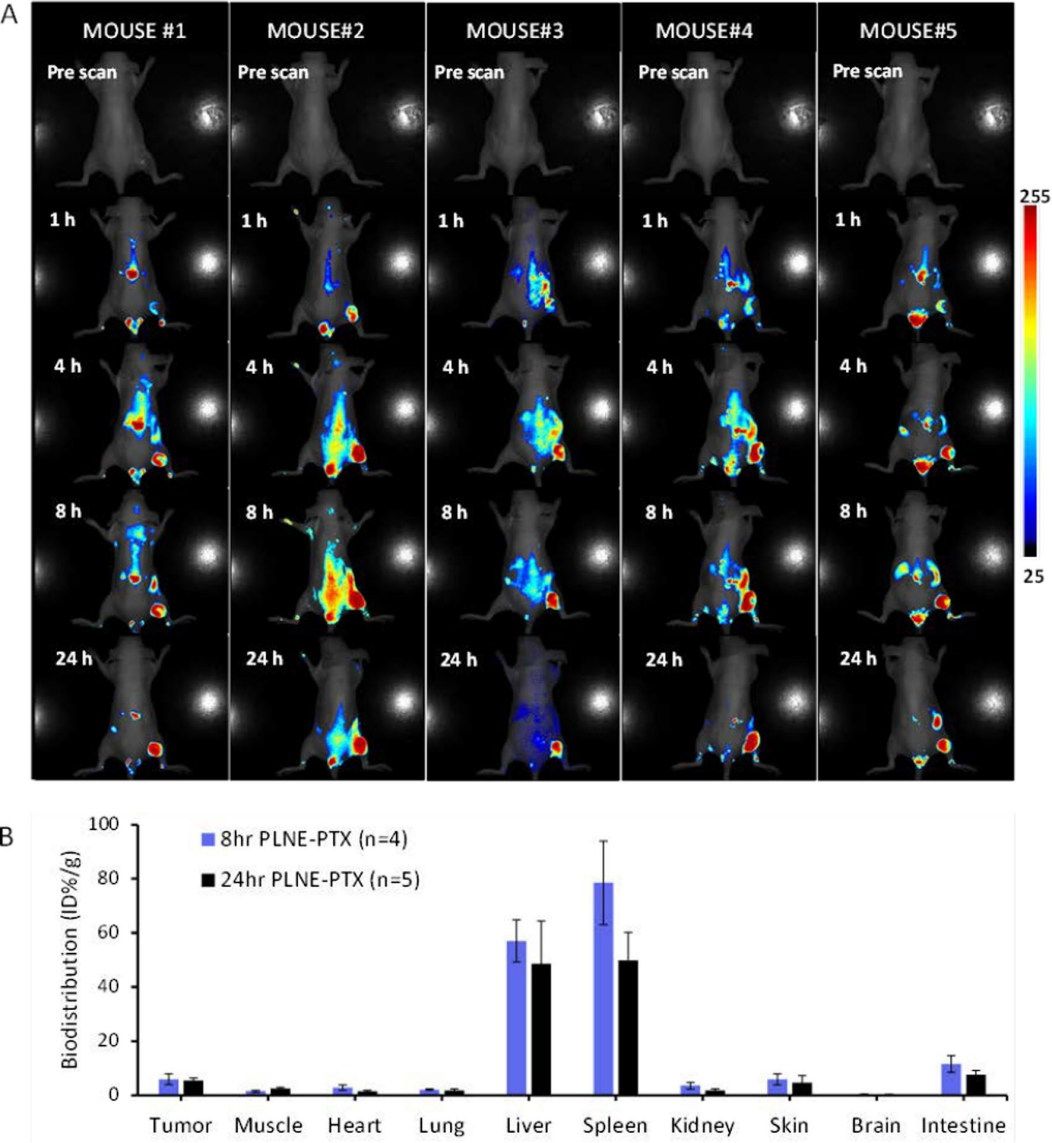
**A** In vivo fluorescence imaging enabled tracking of PLNE-PTX delivery to the tumor within 24 h after drug injection. **B** Biodistribution of PLNE-PTX in organs at 8 h (n = 4) and 24 h (n = 5) post drug injection, respectively

Two-phase exponential decay analysis of the blood clearance of PLNE-PTX revealed that the PLNE-PTX nanoemulsion had a short half-life of t_1/2α_ = 0.52 ± 0.39 h and long half-life of t_1/2β_ = 3.10 ± 0.34 h (n = 5) (Additional file 1: Figure S3). The intrinsic fluorescence nature of porphyrin-lipid enabled real-time tracking of PLNE-PTX delivery to the tumor by Maestro fluorescence imaging system (CRI, USA). As shown in Fig. 6A, PLNE-PTX began accumulating in the tumor 1 h post injection, peaked at 8 h, and remained stable for 24 h (n = 5). Next, tumor accumulation of PLNE-PTX was quantified by fluorescence biodistribution measurements. PLNE-PTX accumulated in the tumor with 5.91 ± 1.99 ID %/g and 5.41 ± 0.89 ID%/g at 8 and 24 h post-injection respectively (Fig. 6B). To ensure the photoreactivity of PLNE-PTX in the tumor, the 24 h post injection timepoint was selected for PDT.

### PLNE-PTX for the combination of PDT and chemotherapy in vivo

**Fig. 7:**
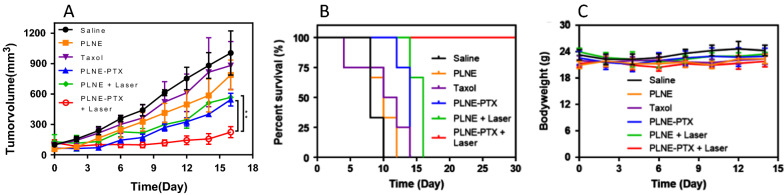
Treatment of KB tumor xenograft-bearing mice with either saline (n = 3), PLNE (40 mg porphyrin-lipid/kg; n = 4), Taxol (7.2 mg PTX/kg; n = 3), PLNE-PTX (7.2 mg PTX/kg, 40 mg porphyrin-lipid/kg; n = 4), PLNE + laser irradiation (10 mg porphyrin-lipid/kg; n = 3), and PLNE-PTX + laser irradiation (1.8 mg PTX/kg, 10 mg porphyrin-lipid/kg; n = 4). Therapeutic efficacy was tracked by **A** tumor volume changes (**represents p < 0.01 by two-way ANOVA test); **B** survival, and **C** body weight measurements

PLNE-PTX was evaluated for combination PDT and chemotherapy in a KB-xenograft mouse model. Mice received either a saline control, empty PLNE (40 mg porphyrin-lipid/kg, a drug delivery agent control), Taxol (7.2 mg PTX/kg, a clinical PTX drug control), PLNE + laser irradiation for single PDT (10 mg porphyrin-lipid/kg), PLNE-PTX for single chemotherapy (7.2 mg PTX/kg, 40 mg porphyrin-lipid/kg), and PLNE-PTX + laser irradiation for combination therapy (1.8 mg PTX/kg, 10 mg porphyrin-lipid/kg). Tumor volumes of all mice were assessed after treatment. The saline group showed exponential tumor growth, reaching a mean tumor volume of 1,004.3 ± 218.2 mm^3^ at day 16 post injection (Fig. 7A). Mice treated with empty PLNE also demonstrated rapid tumor growth, reaching a mean tumor volume of 787.9 ± 147.4 mm^3^ at day 16, suggestive of the minimal effect of empty PLNE on tumor growth. Upon laser treatment, mice injected with PLNE exhibited ~ 44% inhibition in tumor growth with a mean tumor volume of 566.9 ± 8.2 mm^3^ at day 16 post treatment. As such, monotherapy PDT mediated by PLNE was efficacious. As a monotherapy chemotherapy, PLNE-PTX significantly suppressed tumor growth in mice, compared with Taxol at the same PTX dose of 7.2 mg/kg (46% versus 12% in tumor growth inhibition at day 16 with a mean tumor volume of 544.3 ± 62.9 mm^3^ versus 881.4 ± 236.3 mm^3^). When used as combination therapy, treatment of mice with PLNE-PTX at a four-fold lower dose (1.8 mg/kg of PTX) with laser treatment at 50 J/cm^2^ (671 nm laser, 100mW/cm^2^ for 500 s), resulted in ~ 80% inhibition in tumor growth with a mean tumor volume < 120 mm^3^ after 12 days and regrowth to 223.2 ± 54.8 mm^3^ at day 16. The Additional file 1: Figure S4 showed representative photographs in tracking tumor growth over time for each group after treatment.

The greater efficacy of PLNE-PTX combination therapy translated to improved survival, as displayed in Fig. 7B. Mice treated with PLNE-PTX combination therapy had a 100% survival rate on day 24, while all other groups reached endpoint (tumor volume of 500 mm^3^) by day 16 post treatment. No significant body weight changes were observed in any of the experimental groups after treatment (Fig. 7C). Therefore, PLNE-PTX-enabled combination therapy demonstrated superior therapeutic efficacy to either monotherapy PDT or monotherapy chemotherapy. Notably, the reduced PTX dosage in PLNE-PTX combination therapy has potential to reduce PTX-associated side effects.

### PLNE-PTX is a safe multi-functional nanoplatform

**Fig. 8:**
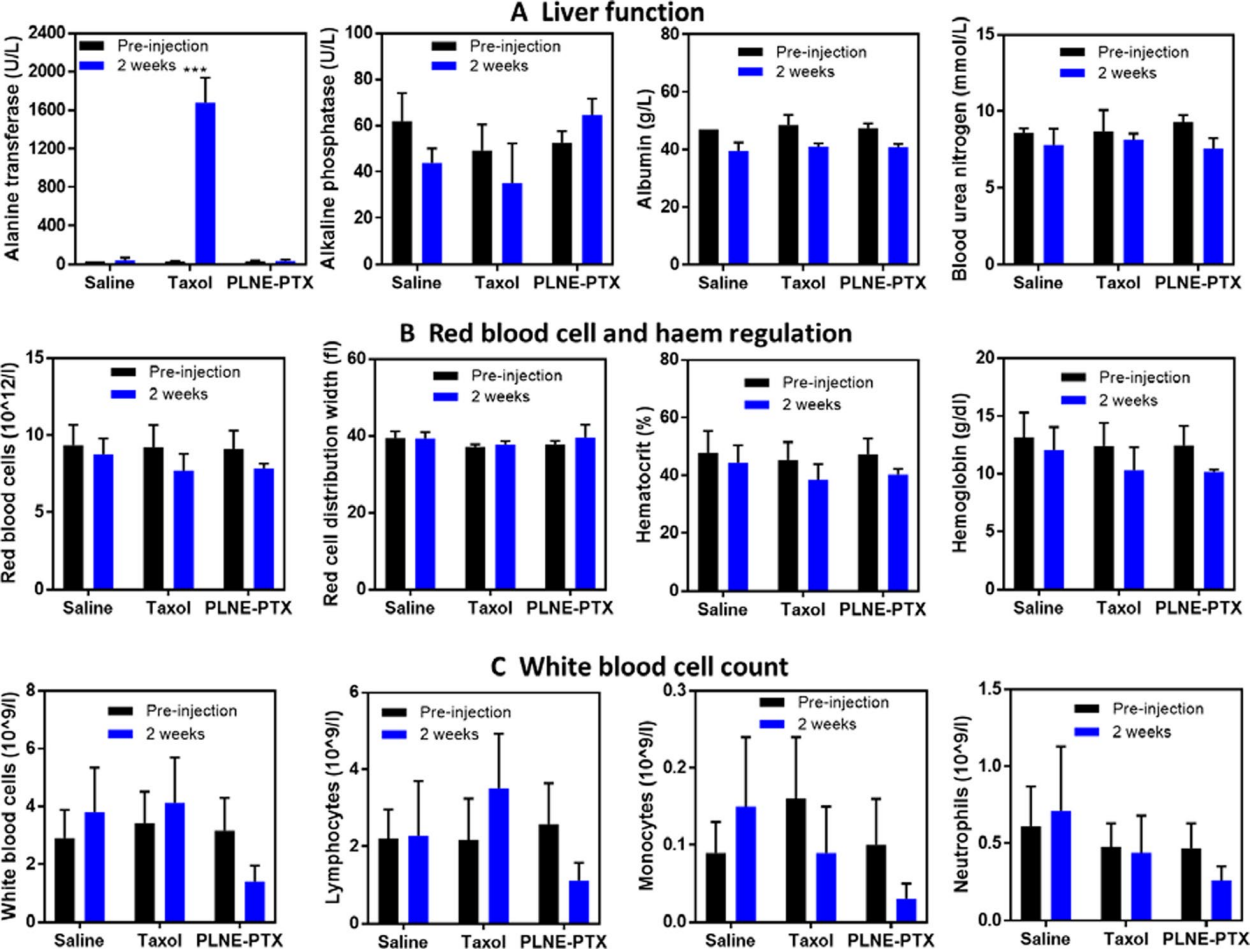
Blood test results for mice prior to and two weeks after intravenous administration of saline (n = 3), Taxol (7.2 mg PTX/kg; n = 4), and PLNE-PTX (7.2 mg PTX/kg, 40 mg/kg porphyrin-lipid; n = 5). (*** p < 0.001)

**Fig. 9:**
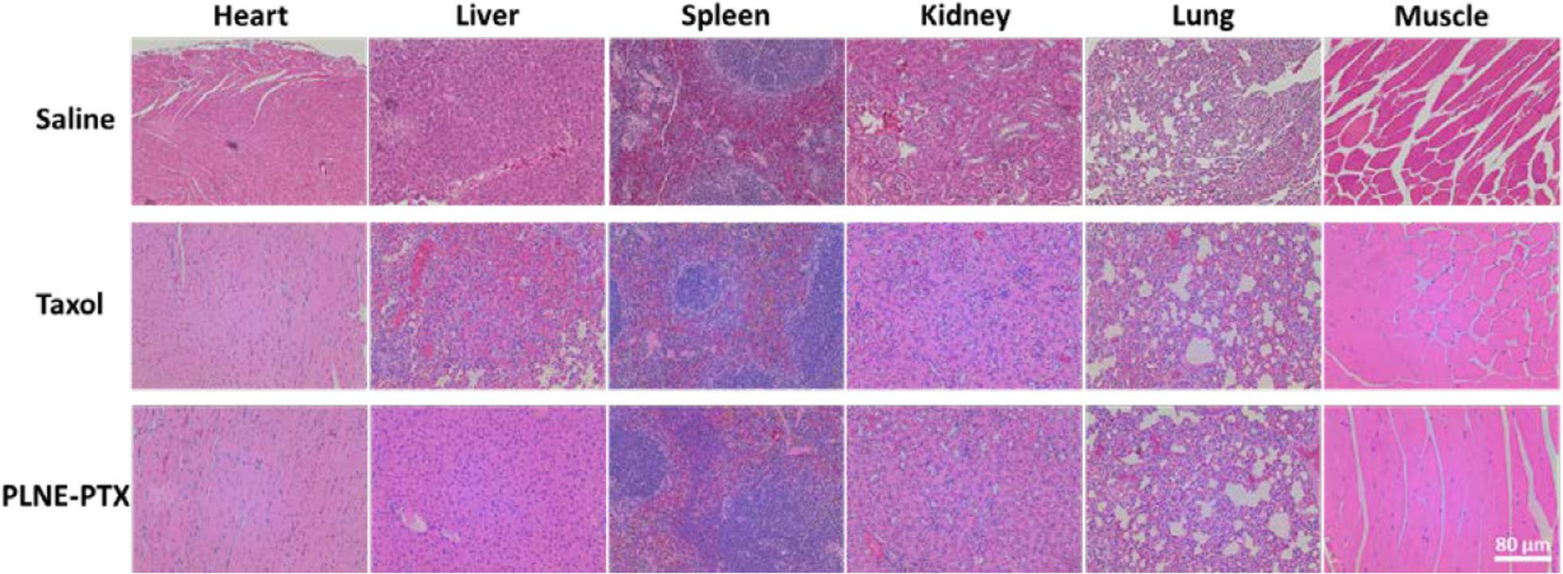
Haematoxylin and eosin stained sections of several organs from mice two-weeks after injection of saline, Taxol (7.2 mg PTX/kg), and PLNE-PTX (PTX dose of 7.2 mg /kg, porphyrin-lipid dose of 40 mg/kg) (scale bar = 80 µm)

The toxicity of PLNE-PTX was assessed by blood tests pre-injection and two weeks post administration and compared to mice treated with saline or Taxol at the same PTX dose (7.2 mg/kg) (Fig. 8A). In mice injected with PLNE-PTX, hepatic function remained normal: no significant changes in alkaline phosphatase, albumin, alanine transferase and blood urea nitrogen were observed. Mice injected with Taxol showed no obvious changes in alkaline phosphatase, albumin, and blood urea nitrogen levels, but had 52.3-fold higher alanine transferase levels (1674.7 ± 261.4 U/L at 2 weeks-post injection *versus* 32.0 ± 6.2 U/L at pre-injection), which may indicate liver damage caused by hepatitis, infection or cirrhosis. By virtue of its unaltered alanine transferase levels, PLNE-PTX may overcome Taxol-induced toxicities related to hepatic function. Red blood cell and hemoglobin levels remained stable for both PLNE-PTX and Taxol groups, indicative of normal physiological regulation of endogenous porphyrin (heme) (Fig. 8B). In addition, white blood cell counts were unaltered for all mice, suggesting that Taxol and PLNE-PTX did not induce significant immunogenic effects (Fig. 8C). This lack of immunogenicity is further supported by histopathology of organs such as the heart, liver, lung, spleen, and kidney, where inflammation was absent, and cellular morphology appeared normal (Fig. 9). Taken together, these toxicity assessments suggest that PLNE-PTX is a safe therapeutic platform.

## Discussion

In this study, a ~ 120 nm PLNE-PTX platform was established as a candidate for combination PDT and chemotherapy. In substitution of porphyrin-salt with porphyrin-lipid and inclusion of a PEGylated lipid to stabilize the nanoemulsion, we generated a biocompatible nanoparticle with a neutral surface (− 2.4 mV), high loading capacity of PTX and porphyrin-lipid photosensitizer, prolonged blood circulation (t_1/2_ = 3.7 h) and excellent accumulation in the tumor (~ 5.4 ID%/g). Compared with other nanoparticle platforms for combination PDT and chemotherapy, PLNE-PTX had a simple composition and was easily synthesized. As such, batch-to-batch consistency was maintained, and production can be scaled-up. In addition, as porphyrin-lipid was enzymatically biodegradable, there is minimal acute toxicity in mice, even with an intravenous dose of 1000 mg/kg [28]. By integrating PDT and chemotherapeutic modalities in a single nanoparticle platform, PLNE-PTX demonstrated clinical potential with its ability to elicit anti-tumor responses at a lower PTX dosage compared to monotherapy chemotherapy and may reduce side effects. This combination strategy may be advantageous for the treatment of advanced-stage cancers where PDT can locally remove primary tumors, while systemic chemotherapy can eliminate deeper-seated tumors and metastases.

The improved therapeutic efficacy of combination PLNE-PTX may arise from additive and potentially synergistic effects of two different mechanisms of tumor cell death. PTX is known to bind to β-tubulin in microtubules, which enhances α-tubulin acetylation and prevents depolymerization, to trigger apoptosis in tumor cells [36–38]. PDT produces singlet oxygen that induces cell death through activation of RIP1 kinase, excessive mitochondrial reactive oxygen species (ROS) production, lysosomal damage, and intracellular Ca^2+^ overload [39, 40]. To assess drug-drug interaction between porphyrin-PDT and PTX, the levels of ROS and α-tubulin acetylation and their interactions can be investigated. In Jérôme Alexandre’s study, PTX promoted ROS generation by enhancing NADPH oxidase activity associated with plasma membranes, and increased extracellular O_2_ and H_2_O_2_ accumulation [41]. Rafah Mackeh et al. reported that ROS exposure to PTX-treated cancer cells further enhanced the level of α-tubulin acetylation [42]. Given these interactions between ROS and α-tubulin acetylation, PLNE-PTX for combination PDT and chemotherapy achieved an additive therapeutic effect. However, through optimization of PDT and chemotherapy dosages, it may be possible to achieve a synergistic anti-tumor effect. In the future, we will examine the levels of ROS and α-tubulin acetylation induced by PLNE-PTX-mediated combination therapy. Further understanding of how interactions between these pathways may enable combination treatment synergy.

Integration of chemotherapy and PDT into a single platform, PLNE-PTX, enables greater spatial and temporal precision of therapeutic agents within the tumor, thereby maximizing e﻿radication. In this study, a significantly enhanced anti-tumor effect was achieved by a single injection of PLNE-PTX, followed by laser irradiation 24 h post-injection. The intrinsic fluorescence of PLNE-PTX enabled real time tracking of nanoparticle accumulation in the tumor, which informed PDT treatment planning. Figure 6 revealed that significant amounts of PLNE-PTX accumulated in the tumor as early as 1 h post-injection, peaked at 8 h, and remained high at 24 h post-injection. With high tumor accumulation of PLNE-PTX between 8 and 24 h, there was a wide window of opportunity for laser irradiation for PDT. We recently demonstrated that subtherapeutic PDT can prime the tumor and facilitate three ~ fivefold enhancement of nanoparticle accumulation in the tumor. Subtherapeutic PDT significantly reduces extracellular matrix density to increase drug accumulation, resulting in improved therapeutic efficacy while decreasing off-target toxicities [43]. Therefore, incorporation of subtherapeutic PDT priming protocol at an early time point (e.g. 1 or 2 h post injection), may further enhance tumor accumulation of PLNE-PTX while reducing off-target toxicities. To enact this strategy, further investigations into light-triggered PTX release and biodistribution of PLNE-PTX at early time points will be necessary. Moreover, light dosage and treatment sequences for subtherapeutic and therapeutic PDT will have to be further optimized.

For enhanced tumor selectivity, PLNE-PTX can be conjugated to targeting ligands [44]. The porphyrin shell can be chelated with radioisotopes (e.g., ^64^Cu) or manganese to allow PET or MRI imaging for cancer detection and treatment planning [29–31]. The oil-core of PLNE can be leveraged to deliver various hydrophobic chemotherapeutics (e.g. camptothecin or curcumin), thus extending its multifunctional modalities.

## Conclusions

A biocompatible and stable porphyrin-lipid nanoemulsion, PLNE-PTX, has been developed. Integrating PDT and chemotherapy modalities, our platform demonstrated superior anti-tumor effects over monotherapies, and holds potential to reduce side effects associated with chemotherapy. PLNE-PTX exhibited nanostructure-driven optical properties: photoreactivity was quenched in intact particles, but efficiently restored upon nanostructure dissociation in the tumor. Thus, our platform is suitable for image-guided treatment planning and holds potential for safe and efficacious combination PDT and chemotherapy.

## Methods

### Materials

Glyceryl trioctanoate was obtained from Sigma Chemical Corporation (USA). 1,2-distearoyl-sn-glycero-3-phosphoethanolamine-N-[amino (polyethylene glycol)-2000] (DSPE-PEG 2000) was purchased from Avanti Polar Lipids (USA). Taxol (PTX 6 mg/mL) was purchased from Haikou pharmaceutical factory CO.LTD (China). Porphyrin-lipid was synthesized accordingly to a previously published method [28]. All other chemicals were of reagent grade. Water was purified with Milli-Q Plus 185 water purification system (Millipore, Bedford, MA). Hoechst 33258 and a LIVE/DEAD® Viability/Cytotoxicity kit were purchased from Invitrogen Corporation (Carlsbad, CA, USA).

### Synthesis of PLNE-PTX

PLNE-PTX was synthesized using a simple sonication method. Briefly, all components: 4 µmol porphyrin-lipid, 0.2 µmol 1,2-distearoyl-sn-glycero-3-phosphoethanolamine-N-[amino (polyethylene glycol)-2000] (DSPE-PEG 2000), 40.8 µmol glyceryl trioctanonate and varying amounts of paclitaxel (PTX) from 0.4 to 1.6 mg (0.5–1.9 µmol), were dissolved and mixed in 3 mL of chloroform (Table 1). Then, the mixture was dried under N_2_ and a lipid film formed. Next, the dried film was hydrated with 6 mL of deionized distilled H_2_O (ddH_2_O) and sonicated in a Branson Ultrasonic Bath Machine at 60 °C for 10 min. The hydrated mixture was further sonicated with a BioRuptor at a low frequency (30 s on/30 s off) for 60 cycles at 25 °C. Nanoemulsions were then stored at 4 °C and characterized.

### Physical and optical properties characterization

Dynamic light scattering (DLS) was used to measure the hydrodynamic diameter and polydispersity index (PDI) of all nanoemulsions. Measurements were performed with a Nanosizer ZS90 (Malvern Instrument) at room temperature. The Zeta potential was measured by the DelsaMax™ Pro (Beckman Coulter instrument). The surface tension of nanoemulsions was measured by the pendant drop tensiometer (Krüss K100). Briefly, a 15 µL drop of porphyrin-lipid NE was created at the end of a 1.80 mm needle. Surface tension was measured at 25 ± 1 °C for several hours in an enclosed chamber to limit evaporation. The morphology of nanoemulsions was imaged with TEM. Samples were negatively stained with 2% uranyl acetate for 2 min. For spectroscopy measurements, PLNE particles were diluted in ddH_2_O (for intact nanoparticle samples) or in ddH_2_O containing 2% Triton X-100 (for disrupted nanoparticles samples). The absorption and fluorescence spectra of the intact and disrupted PLNE were measured, respectively by a UV/Vis spectrophotometer Cary 50 (Agilent, Mississauga, ON) and a Fluoromax-4 fluorometer (Horiba Jobin Yvon, USA) (Excitation: 410 nm, Emission: 650–750 nm, slit width: 5 nm). The following formula was used to assess the fluorescence quenching efficiency:

$${\text{Quenching efficiency }}\left( {\text{\% }} \right) = \left( {1 - \frac{{{\text{Fintact}}}}{{{\text{Fdisrupted}}}}} \right){\text{x }}100{\text{ \% }}$$

where F_intact_ represents the area of the fluorescence peak of intact PLNEs while F_disrupted_ stands for the area of the fluorescence peak of the disrupted PLNEs.

To assess the stability of nanoemulsions in storage at 4 °C, the particle size and PDI of PLNE-PTX samples were monitored weekly. To investigate the serum stability of nanoemulsions, the quenching efficiency of PLNE-PTX samples was measured using a CLARIOstar microplate plate reader (BMG LABTECH) (excitation: 410/8 nm, emission: 671/8 nm, gain = 2500). Fluorescence measurements were obtained from nanoemulsions incubated with 50% serum at 37 °C for 24 h.

### Measurement of PTX encapsulation efficiency and PTX loading capability

After PLNE-PTX synthesis, ultracentrifugation was used to separate unencapsulated drugs from the nanoemulsions. Freshly prepared PLNE-PTX samples with 100 µM porphyrin were ultracentrifuged at 30,000 rpm for 3 h at 4 °C. Particles containing low-density glyceryl trioctanonate oil-core (d = 0.956 g/ml) were concentrated on the top layer of the aqueous solution (d =  ~ 1 g/ml) and the unencapsulated drugs precipitated to the bottom. UPLC-PDA-ELS was used to identify and quantify the components of nanoemulsions. UPLC-PDA-ELS was performed on a Waters Acuity UPLC Peptide BEH C18 column (130 Å, 1.7 μm, 2.1 mm × 50 mm) with a Waters 2695 controller, a 2996 photodiode array detector, and a Waters ELS detector (Waters Canada, Ontario, Canada). The UPLC conditions were as follows: Solvent A) 0.1% TFA and B) acetonitrile; column temperature: 60 °C; flow rate: 0.6 mL/min; gradient from 60% A + 40% B to 0% A + 100% B in 3 min, kept at 100% B for 1 min, followed by a reversion to 60% A + 40% B. The detection wavelengths for porphyrin-lipid and PTX were set at 410 nm and 230 nm, respectively. Glyceryl trioctanoate and DSPE-PEG 2000 were detected by ELS signal. The PTX encapsulation and loading efficiencies of PLNE were calculated with the following formulas:

$${\text{PTX encapsulation efficiency }}\left( {\text{\% }} \right) = \frac{{{\text{Entrapped PTX weight in nanoemulsion }}\left( {{\text{mg}}} \right){ }}}{{{\text{Total weight of feeding PTX }}\left( {{\text{mg}}} \right)}}{\text{x }}100{\text{\% }}$$

$${\text{PTX loading efficiency }}\left( {\text{\% }} \right) = \frac{{{\text{Entrapped PTX weight in nanoemulsion }}\left( {{\text{mg}}} \right)}}{{{\text{Total weight of nanoemulsion }}\left( {{\text{mg}}} \right)}}{\text{ x }}100{\text{\% }}$$

### PLNE-PTX drug leakage profile and intracellular uptake

Dialysis was used to assess PTX leakage. Two mL of PLNE-PTX sample at 300 nM porphyrin was transferred to a 12 K MWCO dialysis tube, and then incubated in 1 L of DPBS with 1% v/v Tween-80 at 37 °C for 72 h with constant stirring at 100 rpm. Tween-80 was used as a surfactant to increase PTX solubility in aqueous solution. After incubation, free PTX was measured by UPLC-PDA-ELS. Fluorescence microscopy was performed to visualize intracellular uptake of nanoemulsions. KB cells were seeded into an 8-well chamber coverglass (Nunc Lab-Tek, Sigma Aldrich, Rochester, NY) at a cell-seeding density of 2 × 10^4^ cells per well. After 24 h culture, cells were incubated with different PLNEs for 6 h. The cells were washed twice with sterile DPBS and incubated with media containing 1 μg/mL of the nuclear stain Hoechst 33,342 (ThermoFisher). Confocal imaging was performed on Leica TCS SP8 STED3X Confocal microscope, using a 63 × oil magnification objective. Hoechst 33342 was detected using the DAPI channel (excitation: 405 nm; emission 410–727 nm). Porphyrin-lipid was excited at 663 nm and emission was collected from 678 to 780 nm. To quantify the uptake of PLNEs, flow cytometry was performed. About 4 × 10^4^ KB cells were seeded per well in a 24-well plate for 48 h. Then, the cells were incubated with PLNE or PLNE-PTX at a concentration of 500 nM porphyrin-lipid for 0–48 h. After incubation, the treated cells were washed with DPBS to remove free nanoparticles. Next, the monomeric porphyrin-lipid fluorescence signal in KB cells was quantified on a Beckman Coulter 153 FC500 five-color analyzer and porphyrin-lipid fluorescence was detected on the APC channel (excitation: 650 nm; emission: 660 nm).

### Evaluation of in vivo pharmacokinetic study

All animal experiments were performed in compliance with University Health Network guidelines. For the pharmacokinetic studies, PLNE-PTX was intravenously injected into healthy female BALB/c mice at a porphyrin-lipid dosage of 10 mg/kg. Approximately 50 µL of whole blood was collected from the saphenous vein pre-injection, and at 5 min, 0.5, 1, 2, 4, 8, 24, and 48 h post-injection. Plasma was separated from whole blood samples by 10 min of centrifugation at 10,000 rpm at 4 °C, and then diluted 200–1000 times by 1% Triton X-100 in DPBS. The fluorescence intensity of monomeric porphyrin-lipid in the plasma was measured by the Fluoromax-4 fluorometer. The α half-life (distribution) and β half-life (elimination) of PLNE-PTX were calculated using a two-phase exponential decay with GraphPad prism software.

### Evaluation of in vivo tumor uptake and biodistribution study

A KB tumor xenograft-bearing mouse model was established according to a previously reported protocol [28]. To determine in vivo tumor uptake of nanoemulsion, animals were intravenously injected with PLNE-PTXs at a porphyrin-lipid dosage of 10 mg/kg when the tumor reached 100–150 mm^3^. In vivo imaging was performed using a Maestro imaging system (CRI, USA) at pre-injection, 1, 2, 4, 8, and 24 h after injection. Fluorescence signal from porphyrin-lipid was obtained using a 546 nm excitation filter and a 700 nm long-pass emission filter. The mice were then sacrificed at 8 or 24 h after injection and several major organs including brain, heart, lung, liver, kidney, spleen, intestine, muscle, and tumor, were dissected. Each organ was homogenized in 2 mL of 1% Triton X-100 in DPBS. PLNE-PTX was extracted and dissociated to unquench the fluorescence of porphyrin-lipid. Homogenized samples were then centrifuged at 13,000 rpm at 4 °C and porphyrin-lipid fluorescence in the supernatant was quantified using Fluoromax-4 fluorometer. A fluorescence-concentration standard curve was also created. Quantitative results are provided as percentage injected dose per gram of tissue (%ID/g).

### Treatment evaluation in vitro

4 × 10^4^ KB tumor cells were seeded per well in 24-well plates. After 48 h, KB cells were incubated with Taxol or PLNE with PTX concentrations ranging from 0–12 nM and porphyrin-lipid concentrations ranging from 0–1200 nM. For cells treated with PLNE-PTX or PLNE-PTX + laser irradiation, to match concentrations of PTX (0–12 nM) and porphyrin-lipid (0–1200 nM), we used a cocktail consisting of 5% PLNE-PTX + 95% PLNE because the optimized PLNE-PTX contained porphyrin-lipid and PTX with a molar ratio of 4.5:1. After 24 h of incubation, treated cells were washed with DPBS to remove free drug. The cells were then exposed to a 671 nm laser at a light dose of 10 J/cm^2^ (Irradiance: 20 mW/cm^2^; exposure time: 500 s). An alamarBlue assay was used to measure in vitro cell viability 24 h after treatment.

### Evaluating therapeutic efficacy in vivo in KB-tumor bearing mice

The therapeutic efficacy of the PLNE-PTX platform was assessed in mice bearing subcutaneous KB xenograft tumors. When tumors grew to100–150 mm^3^, the animals were randomly divided into six groups and treated accordingly: saline control, PLNE (40 mg porphyrin-lipid/kg), Taxol (7.2 mg PTX/kg), PLNE-PTX (7.2 mg PTX/kg, 40 mg porphyrin-lipid/kg), PLNE + laser irradiation (10 mg porphyrin-lipid/kg), and PLNE-PTX + laser irradiation (1.8 mg PTX/kg, 10 mg porphyrin-lipid/kg). At 24 h post intravenous injection, tumors were exposed to a 671 nm laser at a light dosage of 50 J/cm^2^ (irradiance: 100 mW/cm^2^, exposed time: 500 s). The tumor volume and body weight of each animal were measured every two days. A humane endpoint was set for tumor volumes exceeding 500 mm^3^. The tumor volume was calculated from caliper measurements using the formula:

$${\text{Volume}} = \frac{{\left( {{\text{Width}}^{2} {\text{ x Length}}} \right)}}{2}$$

### Evaluation biochemical and hematological blood tests and H&E staining

Comprehensive biochemistry and hematology blood tests were performed in healthy female BALB/c mice to evaluate toxicity of Taxol and PLNE-PTX at a PTX dose of 7.2 mg/kg. Animals were randomly divided into three groups for treatment with saline, Taxol or PLNE-PTX. Blood was collected from the saphenous vein prior to drug administration. Two-weeks after injection, cardiac puncture was employed for blood collection. Blood samples underwent Mammalian Liver Profile tests (Abaxis) and MASCOT hematology profiling (Drew Scientific), according to the manufacturere's protocol. After the sacrifice of mice, organs including the heart, liver, lung, kidney, spleen and muscle were collected for H&E staining. Organs were fixed in 10% formalin, dehydrated with 70% ethanol, and embedded in paraffin. Tissues were cut into 5 µm sections and stained with hematoxylin and eosin and imaged with an Aperio ImageScope.

### Statistical analysis

An two-way ANOVA with Bonferroni correction was used to determine significant differences between experimental groups. P-values < 0.05 were considered significant.

## Supplementary Information

**Additional file 1**: **Figure S1**. Ex vivo tissue fluorescence imaging at 24 h post injection of PLNEnoPEG versus PLNE.** Figure S2**. (A) UV-vis spectrum and (B) fluorescence spectrum of intact PLNE-PTX (ddH2O) or disrupted PLNE-PTX (ddH2O containing 1% Triton X-100).** Figure S3**. Blood clearance curve of PLNE-PTX exhibited short half-life of t1/2α= 0.52 ±0.39 h and long half-life of t1/2β = 3.10 ± 0.34 h (n=5) by two-phase exponential decayanalysis.** Figure S4**. Representative tumor photographs in tracking tumor growth withtime for each group after treatment.

## Data Availability

All data analyzed during this study are included in this published article and its supplementary information files.

## Abbreviations

PTX: Paclitaxel
PDT: Photodynamic therapy
PLNEnoPEG: A simple, two-component porphyrin lipid stabilized nanoemulsion
PLNE: PEGylated porphyrin-lipid nanoemulsion, a favorite version in biological application
PLNE-PTX: Porphyrin-lipid stabilized paclitaxel nanoemulsions
PDI: Polydispersity index
TEM: Transmission electron microscopy
PET: Positron emission tomography
MRI: Magnetic resonance imaging
ROS: Reactive oxygen species
DSPE-PEG 2000: 1,2-Distearoyl-sn-glycero-3-phosphoethanolamine-N-[amino(polyethylene glycol)-2000]
DPBS: Dulbecco's phosphate-buffered saline
ddH2O: Deionized distilled H2O
