# Supplementary material for: Porphyrin-lipid stabilized paclitaxel nanoemulsion for combined photodynamic therapy and chemotherapy
Source: J Nanobiotechnology. 2021 May 25;19:154. doi: 10.1186/s12951-021-00898-1 (PMC8147067; doi:10.1186/s12951-021-00898-1)
Supplement: Supplementary file 1 — Additional file 1: Figure S1. Ex vivo tissue fluorescence imaging at 24 h post injection of PLNEnoPEG versus PLNE. Figure S2. (A) UV-vis spectrum and (B) fluorescence spectrum of intact PLNE-PTX (ddH2O) or disrupted PLNE-PTX (ddH2O containing 1% Triton X-100). Figure S3. Blood clearance curve of PLNE-PTX exhibited short half-life of t1/2α= 0.52 ±0.39 h and long half-life of t1/2β = 3.10 ± 0.34 h (n=5) by two-phase exponential decayanalysis. Figure S4. Representative tumor photographs in tracking tumor growth withtime for each group after treatment. [file 12951_2021_898_MOESM1_ESM.pdf]

Supporting information

Porphyrin-lipid stabilized paclitaxel nanoemulsion for combined photodynamic therapy and chemotherapy

Enling Chang[1,2], Jiachuan Bu[1], Lili Ding[1], Jenny W. H. Lou[1,3], Michael S. Valic[1,2], Miffy. H. Y. Cheng[1], Véronique Rosilio[4], Juan Chen[1], Gang Zheng[1,2,3]**

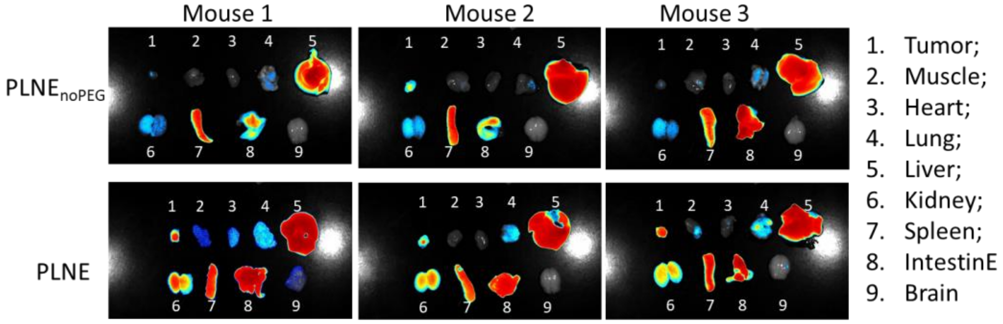

Figure S1. Ex vivo tissue fluorescence imaging at 24 h post injection of $PLNE_{noPEG}$ *versus* PLNE.

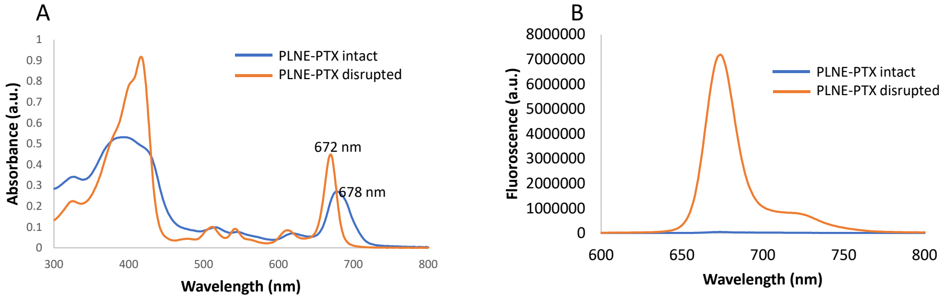

Figure S2. (A) UV-vis spectrum and (B) fluorescence spectrum of intact PLNE-PTX (ddH_2O) or disrupted PLNE-PTX (ddH_2O containing 1% Triton X-100).

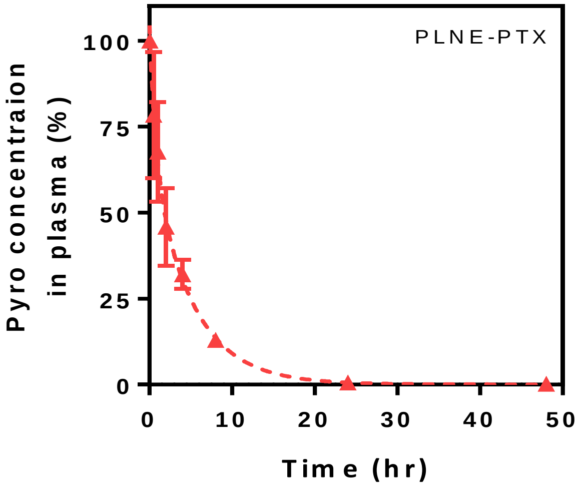

Figure S3, Blood clearance curve of PLNE-PTX exhibited short half-life of t1/2α= 0.52 ± 0.39 h and long half-life of t1/2β = 3.10 ± 0.34 h (n=5) by two-phase exponential decay analysis

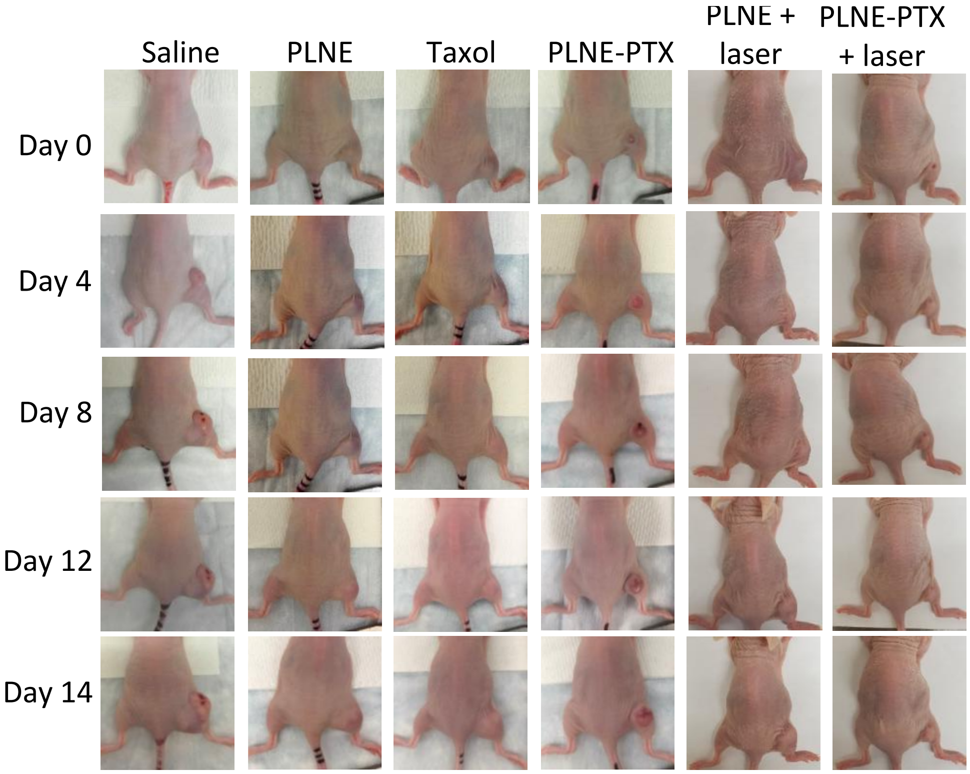

Figure S4. Representative tumor photographs in tracking tumor growth with time for each group after treatment.
